# Foliar Application of Biostimulants Alleviates Salinity Stress in Spinach

**DOI:** 10.3390/plants15081204

**Published:** 2026-04-15

**Authors:** Ömer Taş, Mehmet Ali Cengiz, Hakan Arslan, Deniz Ekinci

**Affiliations:** 1 Department of Agricultural Biotechnology, Faculty of Agriculture, Ondokuz Mayıs University, Samsun 55270, Türkiye; omer.tas@omu.edu.tr; 2 Department of Mathematics and Statistics, College of Science, Imam Mohammad Ibn Saud Islamic University (IMSIU), Riyadh 11623, Saudi Arabia; mamcengiz@imamu.edu.sa; 3 Department of Agricultural Structures and Irrigation, Faculty of Agriculture, Ondokuz Mayıs University, Samsun 55270, Türkiye; hakan.arslan@omu.edu.tr

**Keywords:** tolerance mechanisms, water salinity, ascorbic acid, proline, salicylic acid

## Abstract

Environmental stress factors, especially salinity, are among the most important abiotic stresses that negatively affect plant production worldwide. High salt levels in irrigation water are a major abiotic stress factor that significantly reduces spinach physiological functioning and production, particularly in irrigated areas. Improving the salt tolerance of spinach is critical for sustainable production, and in this study, we tested the hypothesis that exogenous proline (5 µM), ascorbic acid (1 mM), and salicylic acid (1 mM) applications, applied separately, would reduce salinity stress. These applications were performed at regular 14-day intervals starting from the third true leaf stage. For this purpose, plants were exposed to irrigation water salinities of 0.38, 2.0, 4.0, 7.0, 10.0, and 15.0 dS m^−1^, and growth, photosynthetic performance, antioxidant enzyme activities, lipid peroxidation, endogenous proline, and mineral contents were assessed. Increasing salinity to 15 dS m^−1^ decreased leaf area by 53.23% and stomatal conductance by 83.07%, and all these physiological changes were statistically significant. Under salinity conditions, catalase, guaiacol peroxidase, glutathione reductase, glutathione S-transferase, and superoxide dismutase activities increased by 1.13–2.52-fold, while ascorbate peroxidase activity decreased by 59.69%. Malondialdehyde levels increased 6-fold with salinity, indicating enhanced oxidative damage. Consequently, yield decreased by 31% under 15 dS m^−1^ salinity. Although all exogenous applications alleviated salinity stress, the most significant improvement was observed in proline application. Proline increased yield and chlorophyll content by 9% and 8.5%, respectively, and also increased antioxidant enzyme activities by 24.4–66.7%. Salicylic acid treatment increased the K^+^/Na^+^ ratio by 26.6%, and ascorbic acid treatment increased the Ca^2+^/Na^+^ ratio by 36.6%. Overall, low-dose proline application was found to improve photosynthetic pigment content and stomatal conductance, antioxidant defenses, and ion homeostasis in spinach against salinity stress, providing a stronger protective effect compared to ascorbic acid and salicylic acid. Furthermore, it can be concluded that proline application could be an effective way to manage salinity-induced limitations to physiological processes and yields, providing practical applications for sustainable production under saline irrigation conditions.

## 1. Introduction

Common environmental conditions like soil salinity, drought, and heavy metals significantly reduce crop production and other agricultural productivity indicators on a global scale [1,2]. Based on the species of plants and cultivars, recorded yield losses as a result of drought and salt stress vary between 50% and 70% [3]. Salinity is an abiotic stress that inhibits plant growth [4]. The main effects of salinity are poor drainage and waterlogging, resulting in reduced crop yield and farmland productivity [5]. When plants have excessive salt concentrations, increased osmotic stress, ionic imbalance, and deposition of harmful ions occur [6]. Due to Na^+^ toxicity, salt stress negatively affects plant yield and quality by altering physiological and biochemical processes such as reactive oxygen species (ROS) production, which damages vital biomolecules, and disrupts the redox balance [7]. Lipid peroxidation (LPO) results from a reduction in antioxidant properties caused by the increase in ROS levels inside cells. The LPO, also known as malondialdehyde content (MDA), has a significant impact on the permeability and composition of cell membranes [8]. Two types of antioxidant mechanisms counteract the adverse effects of ROS. Enzymatic protection includes superoxide dismutase (SOD), catalase (CAT), ascorbate peroxidase (APX), glutathione reductase (GR), guaiacol peroxidase (GPX), and glutathione s-transferase (GST) enzymes [9], while non-enzymatic protection involves agents [10] such as ascorbic acid (AsA) and phenolic compounds [11].

Transpiration and stomatal conductance are two physiological processes that are reduced by the rise in the ratio of Na^+^ [12]. Increased salt content inhibits the transport and absorption of nutrients (especially Ca^2+^ and K^+^), and salt stress can inhibit photosynthesis [13,14]. Decreasing carbon dioxide intake and restricting photosynthetic pigments, including chlorophyll, reduces the Calvin cycle’s function and indirectly lowers the rate of net photosynthesis [8].

Recently, various plant growth regulators, including salicylic acid (SA) [15], proline (Pro) [16], and AsA [17], have been used to mitigate the detrimental effects of salt stress on plants. AsA has several key roles in controlling responses to abiotic stress. AsA is one of the most studied antioxidants because it is commonly found in all types of plant tissues [18] and has a remarkable role in the antioxidant system by stabilizing ROS production [19]. Pro has been found to trigger growth, physicochemical, and anatomical properties by activating the antioxidant defense mechanism when plants are exposed to salt stress [20]. Exogenous Pro treatment improves plant sensitivity to abiotic stimuli, particularly salt stress, by protecting plants from the destructive effects of ROS by increasing endogenous amounts and intermediary enzymes [21]. In a study on celery, foliar application of Pro increased biomass and chlorophyll fluorescence under salt stress while reducing ROS accumulation and lipid peroxidation and also enhanced antioxidant enzyme activities [22]. SA is a very important regulator that increases plant resistance to abiotic stresses and contributes to the physiological functioning of the plant [14]. It is also important for seed germination, yield, ion absorption and transport [23], gas exchange [24], and regulation of responses to abiotic stresses [25]. Separate application of AsA and SA demonstrated that wheat (*Triticum aestivum* L.) plants growing in salt-affected soils experienced a variety of physiological effects that impacted the qualitative and quantitative parameters of growth and yield [26].

Spinach (*Spinacia oleracea* L.) has gained increasing importance in human nutrition, especially with the development of the food industry [27]. It contains high levels of vitamin C, iron, and calcium. Moreover, it has significant levels of antioxidants, carotenoids, flavonoids, polyphenols, omega-3 fatty acids, and numerous phytochemicals that are beneficial to one’s health [28,29]. Global spinach production is estimated at approximately 31–34 million tonnes per year in 2023–2024 [30]. Spinach is classified as a species with moderate salt tolerance [31]. It has been reported that *Spinacia oleracea* L. can be grown without yield loss at electrical conductivity levels of approximately 8–10 dS m^−1^ in irrigation water under greenhouse and cool-season conditions; however, significant deterioration in growth, photosynthetic pigment content, and ion balance is observed at higher conductivities [31,32]. Spinach is globally important, nutrient-dense, and widely cultivated in regions increasingly affected by water scarcity and salinity. There is a clear need to evaluate its performance under these abiotic stresses; therefore, this study was conducted [27].

This study aims to comprehensively evaluate the effects of exogenously applied Pro, AsA, and SA on the growth, photosynthetic performance, antioxidant enzyme activities, and ion homeostasis of *Spinacia oleracea* L. cv. Matador under salinity stress. While previous studies have demonstrated the protective effects of these three compounds individually against salinity stress in vegetables, a systematic comparison of their combined effects at different salinity levels in *Spinacia oleracea* L. cv. Matador is lacking. Our study aims to identify the most effective strategy and mechanism by comparing the effects of applying these compounds individually at different salinity levels.

## 2. Results

### 2.1. Yield, Dry Weight, and Leaf Area

The interaction of salinity and exogenous applications exerted significant effects on yield, dry weight, and leaf area (*p* ≤ 0.01), indicating that these parameters are highly responsive to salt stress and ameliorative treatments (Table 1).

**Figure 1:**
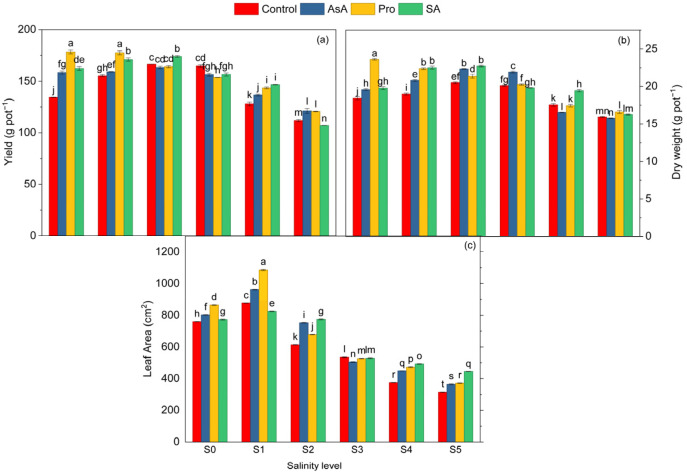
Effect of the application of biostimulants via foliar spray (ascorbic acid, proline, and salicylic acid) and irrigation water salinity interaction on yield (**a**), dry weight (**b**), and leaf area (**c**) in spinach. Different lowercase letters indicate significant differences among treatments based on the LSD test.

Salinity caused a clear dose-dependent reduction in all growth-related parameters. Yield increased slightly at moderate salinity (2–4 dS m^−1^) but declined sharply at higher salinity levels (Table 1). Compared with the control (S_0_), yield was reduced by 31% at 15 dS m^−1^, confirming the strong inhibitory effect of severe salt stress on biomass accumulation (Table 1). AsA, Pro, and SA applications increased the yield by 4%, 9%, and 6.6% compared to the control application, respectively (Table 1). The salinity × exogenous application interaction revealed that the highest yield was recorded under Pro × S_0_, whereas the lowest occurred under SA × S_5_ (Figure 1a). Notably, Pro × S_0_ increased yield by 32.66% compared with C × S_0_, demonstrating that proline enhanced biomass production even under non-stress conditions (Figure 1a).

Dry weight values decreased significantly depending on the salinity intensity, and the most severe decrease was observed at the 15 dS m^−1^ level, showing a 25.80% reduction compared to the control (Table 1). Exogenous applications of AsA, Pro, and SA alleviated the reduction in dry weight, increasing values by 4.9%, 9.2%, and 8.1%, respectively, compared to the control (Table 1). Among these, the Pro application showed the greatest effect, highlighting its superior role in maintaining biomass under stress through enhanced antioxidant protection. Regarding the interaction effects, the highest dry weight was recorded in Pro × S_0_, whereas the lowest value was obtained in the AsA × S_5_ (Figure 1b). Moreover, the Pro × S_0_ increased dry weight by 28.3% compared to the C × S_0_, clearly demonstrating the strong promotive effect of Pro even under non-stress conditions (Figure 1b).

The highest leaf area values were found in plants irrigated with 2 dS m^−1^, but the lowest value was found at 15 dS m^−1,^ with a decrease of 60.11% (Table 1). AsA, Pro, and SA applications increased the leaf area by 10.3%, 15.1%, and 10.4%, respectively, compared to the control application (Table 1). Among these, Pro was the most effective application that alleviated the decrease in leaf area, further supporting its central role in maintaining cellular water balance and sustaining growth under salinity conditions. The highest leaf area was observed in Pro × S_1_, while the lowest value was observed in the C × S_5_ (Figure 1c). Notably, compared to C × S_5_, the SA × S_5_ application increased leaf area by 41.17% (Figure 1c).

### 2.2. Total Chlorophyll, Carotenoid, and Stomatal Conductance

Significant decreases in stomatal conductance, chlorophyll, and carotenoid values were observed with the increase in the irrigation water salinity (*p* ≤ 0.01).

**Figure 2:**
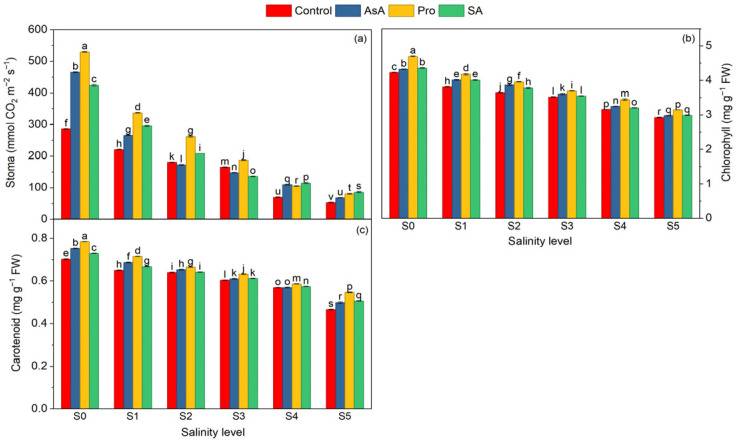
Effect of the application of biostimulants via foliar spray (ascorbic acid, proline, and salicylic acid) and irrigation water salinity interaction on stomatal conductance (**a**), chlorophyll (**b**), and carotenoid (**c**) in spinach. Different lowercase letters indicate significant differences among treatments based on the LSD test.

Stomatal conductance was reduced by 83.06% at the highest irrigation water salinity level compared to the control, which clearly indicates the detrimental effect of salt stress on gas exchange capacity and overall photosynthetic performance (Table 2). Interestingly, exogenous applications alleviated these negative effects to varying degrees, with AsA, Pro, and SA increasing stomatal conductance by 25.98%, 53.98%, and 29.61%, respectively, compared to the control (Table 2). According to interaction data, the highest stomatal value was observed in Pro × S_0_, while the lowest value was observed in C × S_5_. Moreover, compared to C × S0, Pro × S0 increased stomatal content by 85.08% and compared to the C × S5 application, P × S5 by 51.88%, further emphasizing proline’s potential to optimize stomatal performance in addition to stress mitigation (Figure 2a).

The progressive increase in salinity levels resulted in marked reductions in photosynthetic pigments, particularly chlorophyll and carotenoids, highlighting the adverse effects of salt stress on the photosynthetic pathway (Table 2). At the highest salinity level, a 31.59% decrease in chlorophyll content was observed compared to the control (Table 2). Exogenous applications increased the chlorophyll contents in AsA, Pro, and SA applications by 3.4%, 8.5%, and 3.65%, respectively, compared to the control application (Table 2). With regard to salinity × exogenous application interactions, the highest chlorophyll value was observed in Pro × S_0_, demonstrating the inherent stimulatory effect of proline even under non-stress conditions, while the C × S_5_ exhibited the lowest values, reflecting the severe inhibitory impact of high salinity in the absence of protection (Figure 2b). Compared to C × S0, a 10.87% increase in chlorophyll content was found in P × S0 (Figure 2b).

Carotenoid content substantially declined with increasing salinity, and a 32.43% decrease was recorded at the highest salinity level compared to the control (Table 2). Exogenous applications were effective in partially alleviating this decrease, with carotenoid content increasing by 5%, 8.3%, and 3.3% in the AsA, Pro, and SA treatments, respectively, compared to the untreated control (Table 2). With regard to salinity × exogenous application interactions, the highest carotenoid content value was in Pro × S_0_, highlighting the stimulatory effect of proline even under non-stressed conditions, while the lowest value was in C × S_5_, reflecting severe pigment degradation under high salinity without protective supplementation. Moreover, compared to C × S_5_, carotenoid content increased by 17% in Pro × S_5_ (Figure 2c).

### 2.3. Antioxidant Enzymes, MDA, and Endogenous Proline

Enzymatic activities related to antioxidant defense of plants, namely CAT, SOD, GPX, GR, and GST, enhanced significantly in spinach with increasing salinity levels, while APX decreased, compared to the control (*p* ≤ 0.01). The data on CAT, APX, SOD, GPX, GR, and GST enzyme activities of spinach in different irrigation water salinity and AsA, Pro, and SA application conditions are shown in Table 3. Irrigation water salinity and AsA, Pro, and SA applications had significant effects on CAT, APX, SOD, GST, GR, and GPX enzyme activity.

Significant increases were observed in CAT, GPX, GR, and GST activities with rising irrigation water salinity levels. Compared with the control, the CAT, GPX, GR, and GST activity increased by 60.6%, 105.65%, 152%, and 117.1% at the 15 dS m^−1^ level, respectively (Table 3). SOD activity showed a different response, with an increase up to 4.0 dS m^−1^ (13.5% compared to the control) followed by a decline at higher salinity levels (Table 3). In contrast, a significant decrease was observed in APX activity with increasing irrigation water salinity levels. Compared with the control, APX activity decreased by 59.69% at the highest salinity level (Table 3). Furthermore, the application of AsA, Pro, and SA significantly increased CAT, APX, SOD, GPX, GR, and GST activity compared to the control (Table 3). Specifically, the Pro application increased CAT by 56.9%, APX by 54.5%, SOD by 24.4%, GPX by 32%, GR by 66.7%, and GST by 27.3%, compared to the control (Table 3).

**Figure 3:**
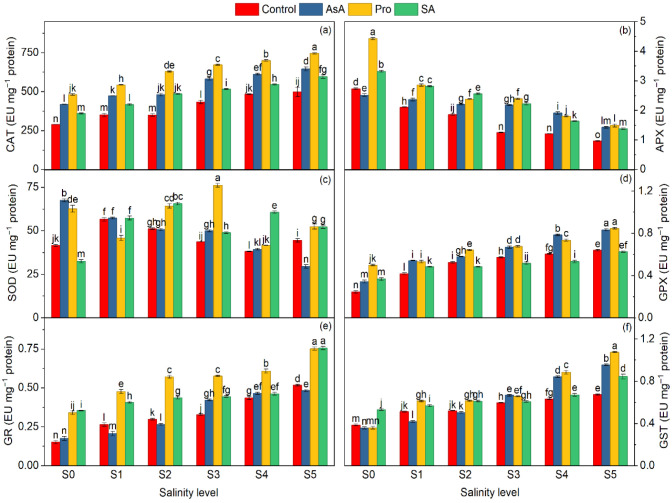
Effect of the application of biostimulants via foliar spray (ascorbic acid, proline, and salicylic acid) and irrigation water salinity interaction on catalase (CAT) (**a**), ascorbate peroxidase (APX) (**b**), superoxide dismutase (SOD) (**c**), guaiacol peroxidase (GPX) (**d**), glutathione reductase (GR) (**e**), and glutathione s-transferase (GST) (**f**) in spinach. Different lowercase letters indicate significant differences among treatments based on the LSD test.

The interaction between salinity and exogenous treatments revealed significant differences in antioxidant enzyme activities. Under severe salinity, Pro × S_5_ revealed the highest activities of CAT, GPX, and GST (Figure 3a,d,f). Conversely, the lowest activities of CAT and GPX were recorded in the C × S_0_, indicating the absence of stress-induced enzyme activation (Figure 3a,d). GR activity reached its maximum with the SA application under severe salinity (Figure 3e). Meanwhile, the lowest GR activity was recorded in the C × S_0_ under non-saline conditions (Figure 3e). The highest activity for APX was found in the Pro application under non-saline conditions, Pro × S_0_, which may indicate that Pro promotes APX activity even in the absence of stress (Figure 3b). However, the lowest APX activity was found in the control under high salinity (C × S_5_), indicating the inhibitory effect of high salt levels on APX (Figure 3b). Moderate salinity effectively stimulated SOD with Pro application, and the highest activity was found in Pro × S_3_ (Figure 3c), but AsA was not sufficient to sustain SOD activity under extreme stress conditions, and the lowest activity was found in AsA × S_5_ (Figure 3c).

Analysis of non-enzymatic stress markers revealed that both proline and MDA contents were significantly affected by salinity (*p* ≤ 0.01).

Increasing irrigation water salinity enhanced proline and MDA content by 502.1% and 40.4%, respectively, at 15 dS m^−1^, compared to the control (Table 4). Exogenous applications, particularly the Pro application, increased endogenous proline levels by 49.92% while simultaneously decreasing MDA by 18.91% relative to the stressed plants without exogenous application (Table 4).

**Figure 4:**
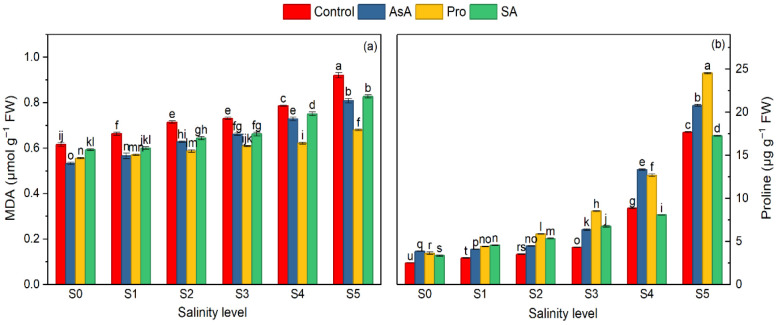
Effect of the application of biostimulants via foliar spray (ascorbic acid, proline, and salicylic acid) and irrigation water salinity interaction on malondialdehyde (MDA) (**a**) and proline (**b**) in spinach. Different lowercase letters indicate significant differences among treatments based on the LSD test.

The interactions between salinity and exogenous application revealed distinct trends. The lowest MDA concentration was recorded in AsA × S_0_, while the highest MDA concentration was observed in C × S_5_ (Figure 4a). Specifically, under severe salinity conditions (15 dS m^−1^), exogenous Pro reduced MDA content by 26.08% compared to the corresponding control (Figure 4a). The lowest proline content was found in C × S_0_, while the highest levels were found in Pro × S_5_ (Figure 4b). Under the highest salinity stress, Pro application increased the endogenous proline content by 38.71% (Figure 4b).

### 2.4. Leaf İonic Homeostasis

Irrigation water salinity and exogenous application had significant effects on leaf Na^+^, Ca^2+^, K^+^/Na^+^, and Ca^2+^/Na^+^ contents (*p* ≤ 0.01).

Ion analysis clearly revealed the negative effect of salinity on spinach ionic homeostasis (Table 5). As expected, Na^+^ has sharply increased the content by 239.62% in 15 dS m^−1^ compared to the control (Table 5). In addition, at high salinity, vital nutrients and homeostasis, K^+^ and Ca^2+^ and their ionic proportions (K^+^/Na^+^ and Ca^2+^/Na^+^) were significantly decreased by 25.31%, 57.32%, 77.87%, and 87.45%, respectively. Exogenous applications of AsA, Pro, and SA were effective in reducing ion imbalances (Table 5). Ascorbic acid and salicylic acid have alleviated salt-induced Na^+^ toxicity, and ionic improvements were particularly remarkable (Table 5). SA reduced Na^+^ accumulation by 14.84%, while increasing K^+^ and K^+^/Na^+^ by 17.8% and 26.6%, respectively, compared to the control (Table 5). AsA increased its Ca^2+^ and Ca^2+^/Na^+^ by 31.2% and 36.6%, respectively (Table 5). AsA was more effective in improving the Ca^2+^ regulation, and SA was more effective in improving the K^+^ balance, while the Pro had a moderate effect.

**Figure 5:**
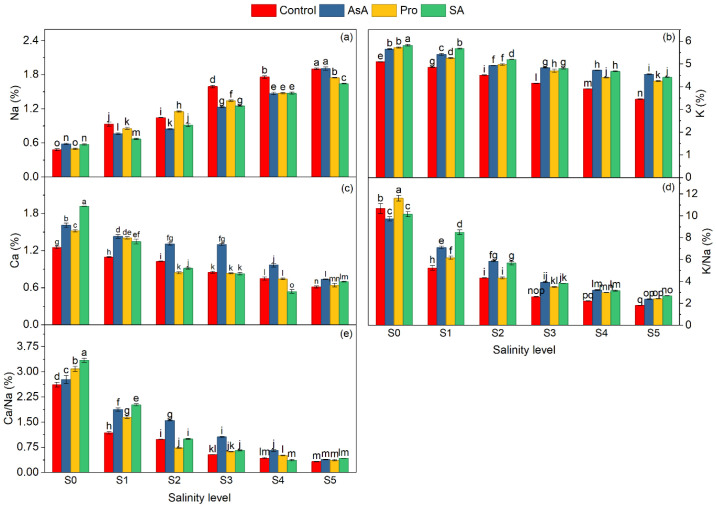
Effect of the application of biostimulants via foliar spray (ascorbic acid, proline, and salicylic acid) and irrigation water salinity interaction on Na (**a**), K (**b**), Ca (**c**), K/Na (**d**), and Ca/Na (**e**) in spinach. Values represent mean ± standard error (n:3). Different lowercase letters indicate significant differences among treatments based on the LSD test.

According to the irrigation water salinity and mineral interaction, the highest Na^+^ content was found in the AsA × S_5_ and the lowest in the C × S_0_ (Figure 5a). The highest K^+^ and Ca^2+^ content were found in SA × S_0_ and the lowest in C × S_5_ (Figure 5b,c). The highest K^+^/Na^+^ and Ca^2+^/Na^+^ ratios were found in Pro × S_0_ and the lowest in C × S_5_ (Figure 5d,e).

### 2.5. Principal Component Analysis (PCA) and Correlation

**Figure 6:**
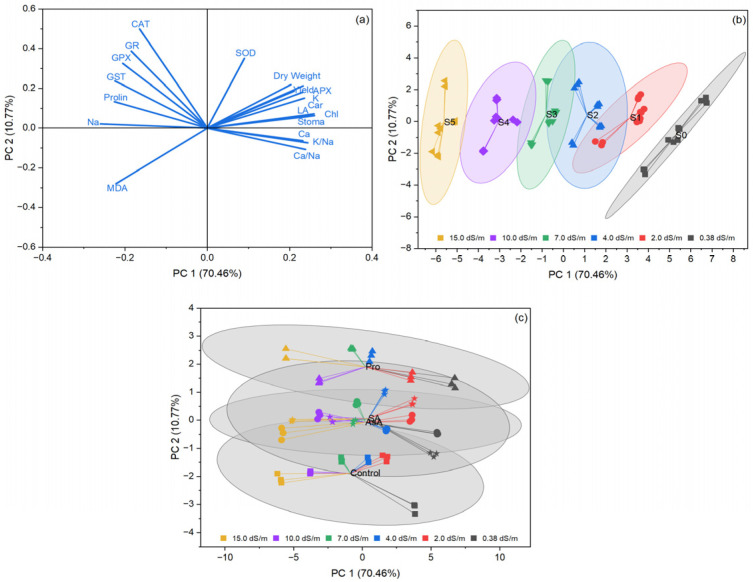
PCA loading plot (**a**), salinity issues (**b**), and relationship between exogenous treatments (**c**) plots showing the effects of irrigation water salinity and exogenous treatment interactions on the evaluated traits of spinach. Abbreviations: APX: Ascorbate peroxidase, CAT: Catalase, GPX: Guaiacol peroxidase, GR: Glutathione reductase, GST: Glutathione s-transferase, SOD: Superoxide dismutase, MDA: Malondialdehyde, Car: Carotenoid, Chl: Chlorophyll, LA: Leaf area.

PCA was performed to evaluate the role of exogenous application of AsA, Pro, and SA in reducing salt stress in spinach for 19 parameters. MDA, proline, CAT, GR, GPX, and GST were clustered at the same location in PCA (Figure 6a). Yield, APX, Ca^2+^/Na^+^, K^+^/Na^+,^ and stomatal conductance were clustered in the same area (Figure 6a). Irrigation water salinity issues are also clustered separately, as seen in Figure 6b. Exogenous applications also differentiated Pro from SA, AsA, and the control, while SA and AsA clustered together (Figure 6c). All exogenous treatments (AsA, Pro, and SA) were clustered in the same areas, responding to salinity stress (Figure 6c).

**Figure 7:**
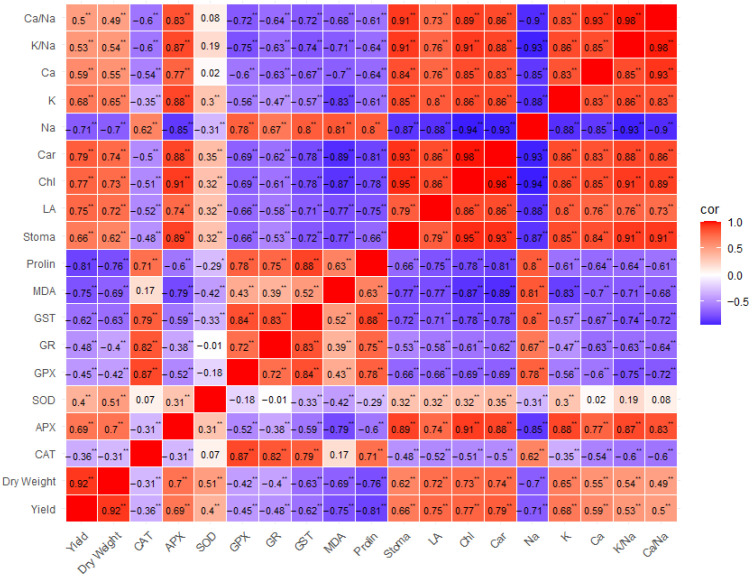
Correlation plots showing the effects of irrigation water salinity and exogenous treatment interactions on the evaluated traits of spinach. Abbreviations: APX: Ascorbate peroxidase, CAT: Catalase, GPX: Guaiacol peroxidase, GR: Glutathione reductase, GST: Glutathione s-transferase, SOD: Superoxide dismutase, MDA: Malondialdehyde, Car: Carotenoid, Chl: Chlorophyll, LA: Leaf area. **: Indicates significance at the 1% level; *: Indicates significance at the 5% level

According to the correlation table, yield was positively correlated with carotenoids and K^+^, while it was negatively correlated with MDA, proline, and Na^+^ (Figure 7). Antioxidant enzymes GST and GR showed a negative correlation with yield, while APX showed a positive correlation. SOD and GPX showed a moderate correlation (Figure 7). Na^+^ was positively correlated with proline, MDA, GST, and GPX, while it was negatively correlated with APX (Figure 7). K^+^ showed a highly positive correlation with carotenoids, chlorophyll, and stomata (Figure 7). K^+^/Na^+^ showed a highly positive correlation with chlorophyll and stomatal conductance, while it showed a negative correlation with GPX, GST, and MDA (Figure 7). A positive correlation of yield with leaf area was also associated with increased yield (Figure 7). No significant correlation was found between SOD and CAT, POD, GR, Ca, K/Na, Ca/Na, and between MDA and CAT. Highly significant correlations were determined between the other parameters (Figure 7).

## 3. Discussion

Salinity stress is the main abiotic stressor with detrimental effects on crop production and quality because it causes a considerable decline in plant physiological and morphological characteristics [33]. Salinity is known to profoundly affect plant physiology through both osmotic and ionic stress. High salt concentrations in the root zone first reduce leaf water potential and turgor, leading to decreased cell expansion and reduced leaf area development [34,35]. Under osmotic stress, stomatal conductance and transpiration typically decrease, restricting CO_2_ entry into the leaf. This reduction in CO_2_ diffusion leads to a lower net photosynthetic rate, decreased chlorophyll content, and a decline in PSII efficiency [34].

Specifically in *Spinacia oleracea* L., salinity reduces yield, leaf area, stomatal conductance, chlorophyll, and carotenoids while increasing Na^+^ accumulation, oxidative stress markers, and antioxidant enzyme activities [36,37]. Salt stress significantly impairs plant development by imposing osmotic restrictions that limit the uptake of nutrients and water while also inducing ionic toxicity by disrupting cellular mineral homeostasis [38]. The detrimental impacts of salinity might be reduced by taking quick actions to increase crop development and yield [39]. One of these methods is the use of particular agronomic controls, or the exogenous application of various biostimulants [40]. Exogenous application has previously shown improvement in plant growth parameters and protection against the detrimental effects of salinity stress [41].

Irrigation with varying salt concentrations (15 dS m^−1^) negatively affected spinach yield and leaf dry weight by disrupting ionic homeostasis and causing osmotic stress. In general, increased Na^+^ in the soil causes accumulation of the element in plant tissues, inhibiting photosynthesis, enzymatic activities in the cytoplasm and chloroplast, and causing ionic degradation, leading to reduced plant growth [42]. While spinach tolerated salinity up to 4 dS m^−1^ without yield and dry weight loss, a significant decline was recorded under 15 dS m^−1^ stress conditions (Table 1). This decline is consistent with the osmotic and ionic effects of salinity, which restrict water and nutrient uptake, impair photosynthetic efficiency, and disrupt metabolic homeostasis, thereby limiting biomass production [43]. Previous studies have similarly demonstrated that high salt concentrations reduce water availability and disrupt ionic balance, limiting photosynthetic efficiency and growth [44,45]. Exogenous applications of AsA, Pro, and SA significantly mitigated the unfavorable impacts of salt stress on yield and dry weight, with the best result achieved from Pro (Table 1). These compounds individually mitigate the osmotic and oxidative stress caused by salinity, protecting cellular metabolic functions and increasing yields under saline irrigation [46]. In many plants, foliar-applied proline, as a biostimulant, increases yield under salt stress by maintaining ion and water homeostasis, supporting photosynthetic mechanisms, and strengthening antioxidant defenses [22,47]. These findings are supported by similar studies in different plants showing that salinity stress causes losses in spinach yield, but exogenous applications (especially Pro) can significantly reduce these losses by regulating physiological and biochemical tolerance mechanisms [48,49].

Decreased leaf area is one of the most frequent reactions to salt stress [50]. This study observed a significant decrease in leaf area as irrigation water salinity increased (Table 1). This decrease can be associated with ionic imbalance and osmotic stress [51]. This effect of salt stress may have restricted water uptake and nutrient retention, thus causing cell expansion and deterioration of physiological functions. Ors and Suarez (2016) [32] reported that leaf area in spinach decreased with increasing salinity (7–15 dS m^−1^). Similarly, Wang et al. (2023) [52] reported that increasing salinity negatively affected leaf area. However, Pro has been shown to ameliorate this effect by increasing salt tolerance in plants [53]. In our study, the effects of salt stress were reduced by separate exogenous applications of Pro and AsA. Overall, these results indicate that salinity significantly restricts leaf area development, but exogenous supplements can mitigate these effects and significantly improve leaf area under salt stress conditions, thereby enhancing plant tolerance to salt-induced osmotic stress. Leaf area expansion increases chlorophyll concentration, indicating that plants are absorbing nutrients and water regularly [54]. Similar protective effects of exogenous treatments against salinity have been documented in *Lactuca sativa* L. and *Prunus amygdalus* L., confirming the consistency of the present findings with earlier research [55,56].

Salt stress causes excessive ROS production, mostly in chloroplasts [57], which negatively affects photosynthesis by interfering with electron transport, chlorophyll degradation, membrane integrity, and proteins involved in photosynthesis, yielding results similar to our study [58]. This may also negatively affect photosynthesis by simultaneously impairing stomatal function and chlorophyll activity [59]. Stomatal closure and reduced photosynthetic rates in plants have also been implicated in K^+^ deficiency [60]. K^+^ plays a key role in guard cell turgor, CO_2_ diffusion through stomata and mesophyll, and the activation of photosynthetic enzymes, so its deficiency restricts CO_2_ supply and biochemical capacity, leading to lower photosynthesis [61,62]. Exogenous osmoprotectant applications help reduce the unfavorable effects of salinity by regulating stomatal conductance and controlling photosynthesis [63]. The effect of exogenous application of Pro on chlorophyll content may be associated with the stabilization of photosynthetic reactions, possibly through improved osmotic balance and protection of pigment–protein complexes under saline conditions [64]. These findings suggest that antioxidant- and osmolyte-mediated protective mechanisms can partly alleviate salinity-induced pigment loss [65]. Also, our results revealed that the treatments might play a strong role in stabilizing pigment-protein complexes and maintaining antioxidant defense under salinity [66]. These data demonstrate that although salinity imposes strong constraints on stomatal, chlorophyll, and carotenoid levels, exogenous applications, particularly of Pro, can significantly modulate these effects, thus contributing to improved water relations and photosynthetic stability in saline environments. In this process, proline primarily functions as a compliant osmolyte and ROS scavenging molecule, maintaining cell turgor and ionic homeostasis, and protecting chloroplast membranes, photosynthetic pigments, and photosystem activity under salinity stress [64]. Many research findings have reported that especially exogenous application of Pro significantly increases the chlorophyll, carotenoid, and stomatal values in plants under salt stress by stimulating their biosynthesis or inhibiting their degradation [67,68].

Antioxidant activity was greatly increased by salinity stress, indicating a defensive strategy in stressed plants [69]. In this study, the increase in CAT, GR, GST, and GPX activities observed with increasing irrigation water salinity demonstrates that the ROS detoxification mechanism is systematically upregulated [70]. Whereas GPX and CAT enzymes detoxify hydrogen peroxide, SOD is an enzyme that catalyzes the dismutation of superoxide radicals into regular molecular oxygen and hydrogen peroxide [71,72]. In the current study, CAT, GR, GST, and GPX activities were found to be enhanced under increasing irrigation water salinity (Table 3). At moderate salinity, the increase in SOD activity suggests balanced regulation of ROS without significant membrane damage (Table 3). It has been reported that SOD activity in spinach performs a primary defense function up to a threshold of 4.0 dS m^−1^ (Table 3). Beyond this point, a decline in SOD activity indicates that the plant’s enzymatic detoxification capacity is exceeded by severe salt stress, leading to failure in ROS signaling [44]. There are many studies showing that salt stress increases the activities of many enzymes, such as *Daucus carota* L. (GPX) [49], *Sorghum bicolor* (CAT) [73], *Phaseolus vulgaris* L. (GST) [74], *Saccharum officinarum* L. (GR) [75], and *Oryza sativa* L. (SOD) [76], in various plants. Studies have generally reported that APX activity increases with increasing salinity stress [77]. However, this activity was found to decrease in our study. This decrease may be due to its sensitivity to low ascorbate levels and its increased susceptibility to inactivation by thiols and protein inhibitors [78]. The decrease in APX activity under salinity stress reported in this study is in line with some previous reports documenting a similar pattern [79,80]. The varied reactions of antioxidant enzymes to salt stress demonstrate the complexity of the plant defense system [81]. Some enzymes are strongly induced to mitigate oxidative damage, while others are suppressed under severe stress, indicating a defect in ROS detoxification [82].

The use of various osmoprotectants improves the antioxidant response to stress and increases enzyme activity, assisting plants in maintaining membrane structure and osmotic adjustment [83]. Exogenous applications play an important role in increasing the resistance mechanism against stress in plants by supporting the antioxidant defense system [84]. These findings imply that enzymes are crucial components of spinach’s defensive mechanism by facilitating the reduction of superoxide to H_2_O_2_ and O_2_. Increases in the activity of the antioxidant enzymes such as CAT, GR, GST, APX, SOD, and GPX were detected in spinach with all exogenous applications. Due to their antioxidant properties, biostimulants not only directly neutralize ROS but also aid in the synthesis of antioxidant enzymes, which ultimately reduces MDA content. Additionally, biostimulants are known to influence the accumulation of osmotic protectants and secondary metabolites, which in turn contribute to oxidative stress tolerance [81]. Among the applications, the highest activity was found in the Pro application compared to the control. More importantly, the results highlight that exogenous Pro not only regulates enzyme activity but also significantly supports the antioxidant defense system, playing a stronger protective role compared to other treatments. The scavenging of ROS by antioxidant enzymes and cellular antioxidant compounds may be more efficient due to the effect of Pro [19]. Low-dose proline may offer a unique strategy for strengthening enzymatic defenses and maintaining redox homeostasis in spinach under salinity stress. Increases in the activities of antioxidant enzymes such as CAT, GR, GST, APX, SOD, and GPX in spinach in all exogenous applications supported our findings with studies conducted on many different plants [21,85,86].

H_2_O_2_ and MDA formed under salt stress damage the cell membrane system and cause oxidative stress, which affects lipid peroxidation [87]. As MDA content is a key indicator of ROS-induced cellular damage, the substantial increase observed in our study is a direct reflection of the severity of salt-induced membrane degradation [88]. The results of this study are consistent with the results of many researchers; a significant increase in MDA content was observed under salinity, indicating increased membrane lipid peroxidation [89]. Exogenous applications significantly reduced MDA levels by increasing antioxidant enzymes such as SOD, GPX, and CAT, which play an important role in removing increased ROS in the saline environment and maintaining cell homeostasis. AsA, Pro, and SA applications revealed that they reduced the MDA content. In addition, it was found that Pro was the best exogenous application to reduce the MDA content (Table 4). Pro probably acts as a metabolic shield, stabilizing the phospholipid bilayer against attack by ROS and preventing the initiation of the lipid peroxidation chain [85]. Similar results were found in studies conducted on different plants regarding these practices to protect plants and to support our findings [90,91].

Plants act as protectors against osmotic stress by producing large numbers of osmolytes such as proline under high salinity stress [92]. Although the accumulation of proline is a well-documented physiological response to salt and drought stress, its role in our study goes beyond simple osmotic adjustment [93]. Proline acts as a multifunctional defense coordinator that removes ROS to protect proteins, cell membranes, and vital cellular structures from oxidative degradation [94,95]. We observed that endogenous proline levels increased significantly at high salt concentrations, in line with patterns observed in other salt-tolerant species [63]. However, exogenously applying Pro further amplified this accumulation, resulting in the highest values among all treatments. This suggests that exogenous Pro not only supports internal balance but also minimizes oxidative effects and reduces cell death rates by stimulating the plant’s own biosynthetic pathways [96]. By limiting water loss and reducing harmful ion concentrations, proline effectively bridges the gap between osmotic balance and antioxidant defense, a beneficial interaction that has been corroborated across various plant species [97,98].

One of the most important processes in salt stress is thought to be ion homeostasis imbalance [99]. Under high salinity, the accumulation of phytotoxic Na^+^ leads to a reduction in the availability of K^+^ and Ca^2+^, signaling a failure in the plant’s ion selectivity mechanisms and resulting in a severe nutritional imbalance [100]. Due to high salt accumulation by roots in the rhizosphere, Na^+^ is easily absorbed by plants, and the K^+^ level decreases [101]. As a result, in line with our study, a decrease in the K^+^/Na^+^ ratio occurs, which leads to the deterioration of physiological functions necessary for growth [97]. In contrast, many plants defend against salinity stress by eliminating Na^+^ uptake and balancing the optimum K^+^/Na^+^ ratio by taking in more K^+^. In addition, exogenous applications have shown a positive relationship with resistance to stress conditions and improvement of growth parameters by providing high K^+^, Ca^2+^, and K^+^/Na^+^ ratios in plants [38,102]. Pro can help stabilize the K^+^/Na^+^ ratio. It does this by restricting the transport of toxic ions. It also promotes K^+^ retention. This positive balance of ions, along with less oxidative damage, makes sure that the cell’s machinery can still do its job even when there is a lot of salt in the system [48]. Such improvements in ion balance are vital to eliminate the negative effects of salinity on physiological processes and plant growth. Biostimulant molecules such as proline, salicylic acid, and ascorbic acid enhance salinity tolerance by reducing Na^+^ and the Na^+^/K^+^ ratio, improving K^+^ and other nutrient uptake, and strengthening antioxidant defenses, thereby maintaining ion homeostasis and cellular integrity under salt stress [73,103].

## 4. Materials and Methods

### 4.1. Experimental Site, Growth Conditions, and Plant Source

**Figure 8:**
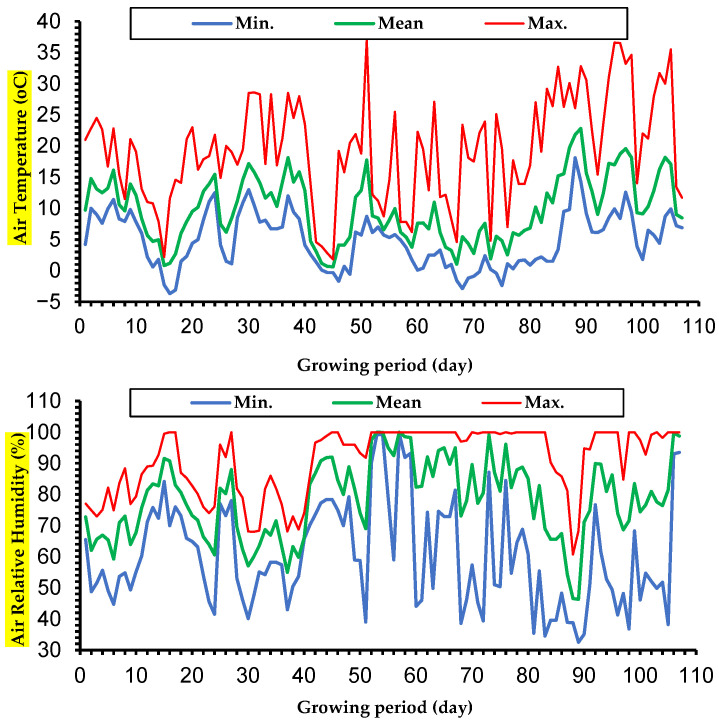
Daily values of air relative humidity and temperature during the growth season.

The study was carried out between 4 January and 20 April 2022 at Samsun Ondokuz Mayıs University Agricultural Research Center under a rain shelter. An electronic data recorder (LYK-20E Data Logger, LOYKA Company, Istanbul, Türkiye) was used to collect variations in Daily air temperature and air relative humidity values during the experimental period. The meteorological data are depicted in Figure 8. The relative humidity varied from 33 to 100% (average 75.5%), while the air temperature varied from −3.7 to 36.6 °C (average 12.16 °C), respectively.

*Spinacia oleracea* L. cv. Matador was used as the plant material. After the seeds were sterilized, they were prepared for sowing. After sterilization, seeds were sown to a depth of 2 cm at 14 seeds per pot. After emergence, thinning was done to 3 plants in each pot. The nutrients required by spinach have been determined by soil analysis, and fertilization was performed based on this analysis. Before the sowing, 1.10 g of diammonium phosphate fertilizer per pot was applied to all pots as a phosphorus source, and 1.25 g of urea was used as a nitrogen source at two different times (pre-planting and 4–6 leaf period).

### 4.2. Preparation of the Soil

A soil sample was obtained from 0 to 25 cm depth at Ondokuz Mayıs University Agricultural Research Center, Samsun, Türkiye. The experimental soil was classified as sandy loam, with a pH of 7.03 and an electrical conductivity of 0.51 dS m^−1^. The soil contained 1.86% organic matter and 13.74% CaCO_3_, with total nitrogen and available phosphorus contents of 0.09% and 17.25 mg kg^−1^, respectively. The exchangeable cation contents were 24.26 cmol kg^−1^ Ca^2+^, 7.52 cmol kg^−1^ Mg^2+^, 0.56 cmol kg^−1^ K^+^, and 0.47 cmol kg^−1^ Na^+^.

### 4.3. Experimental Design, Irrigation Water Salinity, and Foliar Treatments

The present work was performed in a total of 72 pots [6 salinity levels × 4 exogenous applications × 3 replicates = 72 pots]. Then, 25 kg of air-dried and sieved (4 mm) soil was added uniformly into the pots (32 cm high and 33 cm inner diameter). Irrigation water salinity [S_0_ (0.38 dS m^−1^)] was used as a control application by adding two different salt types (NaCl and CaCl_2_), S_1_ (2.0 dS m^−1^), S_2_ (4.0 dS m^−1^), S_3_ (7.0 dS m^−1^), S_4_ (10 dS m^−1^), and S_5_ (15 dS m^−1^). Salinity concentrations were determined according to the study of Kim et al. (2021) [42]. Saline nutrient solutions for S_1_, S_2_, S_3_, S_4,_ and S_5_ were created by adding NaCl/CaCl_2_ at the rates of 0.50/0.50, 1.20/1.21, 2.38/2.34, 3.54/3.54, and 5.18/5.21 g L^−1^, respectively, to reach the desired salinity levels. To ensure the correct use of specified salinity levels, different saline nutrient solutions were prepared separately in 100-liter tanks.

Each pot’s soil was saturated with tap water, and the pot’s surface was then covered with plastic bags to restrict evaporation. Each pot’s soil water content was measured when drainage was completed, and field capacity was assumed. All pots were irrigated with non-saline water until stabilization. Irrigation water salinity applications are started on the 50th day after sowing. The pots were constantly weighed, irrigation was performed when the field reached capacity, and 30% of the usable water was depleted.

Three biostimulants were used for correction, and to standardize foliar application, distilled water was applied to the control to avoid favoring the treatments with diluted biostimulants. Four different exogenous applications were administered via solutions, including (1) distilled water (C), (2) 1 mM (AsA), (3) 5 µM (Pro), and (4) 1 mM (SA). The application concentrations were determined based on the results of previous studies of *Vicia faba* L. treated with 0.5 mM AsA and Pro and *Punica granatum* L. treated with 1 mM SA under saline stress conditions [104,105]. AsA, Pro, and SA exogenous application solutions were prepared with 0.01% Tween 20, deionized water, and DMSO (dimethyl sulfoxide), and these applications were made manually using a hand sprayer. Pots subjected to different exogenous applications were covered with plastic sheeting to prevent mixing of applications. Foliar applications were applied to the leaf surface of each plant, with amounts varying depending on the plant’s growth status, ranging from 10 to 30 mL per plant. Foliar applications began 7 days after the first salinity application and continued with 14-day intervals. A total of 4 applications were carried out during the research. Exogenous applications continued until all leaves were wet. Spinach was harvested 7 days after the last exogenous application.

### 4.4. Yield and Dry Weight Analysis

Three plants from each pot were harvested 106 days after planting, and yield was measured with a precision balance (Precisa, XB-620M, Zurich, Switzerland). All harvested plants were then dried in an oven (Nüve, ES-500, Ankara, Türkiye) at 70 °C for approximately 72 h until completely dry. The dried plants were weighed using a precision balance (Precisa, XB-620M, Switzerland) to determine their dry weight.

### 4.5. Leaf Area Analysis

Whole harvested spinach leaves from each plant were photographed and analyzed using Adobe Photoshop CS6, 2026. (Adobe Systems Inc., San Jose, CA, USA) imaging software. Leaf area was calculated using the obtained data.

### 4.6. Leaf Chlorophyll Content, Carotenoid, and Stomatal Conductance

To determine the pigment contents, 0.1 g of fresh leaf sample, 0.05 g of MgO (magnesium oxide), and 0.125 g of quartz sand were crushed and homogenized under optimum conditions by adding 80% (*v*/*v*) acetone in a porcelain mortar. The resulting homogenate was then filtered and made up to 25 mL with acetone. The prepared tubes were then centrifuged at 10,000× *g* for 15 min. The absorbance of the supernatants obtained after centrifugation was measured with a spectrophotometer device (Shimadzu UV-1800, Kyoto, Japan) at wavelengths of 480, 645, and 663 nm. Total chlorophyll and total carotenoid values were obtained from fresh leaves as mg g^−1^ fresh weight. Total chlorophyll and carotenoids were calculated using the following formula [106,107].

Total chlorophyll (mg g^−1^ fresh weight) = [(20.2 ×A645) + (8.02 × A663)] × V/(1000 × w)

Carotenoid (mg g^−1^ fresh weight) = (A480 × V)/(250 × w)

where

A663 = absorbance value at 663 nm;

A645 = absorbance value at 645 nm;

A480 = absorbance value at 480 nm;

V = volume of extract;

w = fresh leaf weight.

The leaf stomatal conductance was determined in three separate regions of the fully matured upper leaves of the plants in each pot using a porometer (AP4 Porometer Delta-T, Cambridge, UK), and the average value was calculated in mmol m^−2^ s^−1^.

### 4.7. Plant Minerals Analysis

At the end of the study, all plants in the pots were harvested, and the fresh plant weight was recorded. The plant samples were then dried by holding them at 70 °C for 48 h. The dry plant weight was recorded. The filtrate from these dried plants was obtained by dry combustion, and the levels of the Na^+^, Ca^2+^, and K^+^ minerals were determined using a flame photometer; unit = % [108].

### 4.8. Activity of Antioxidant Enzymes Analysis

Four leaves per treatment were collected at the conclusion of the experimental process and quickly frozen at 80 °C for the purpose of measuring CAT, APX, SOD, GR, GST, and GPX activities. For preparation of the homogenate, 1 g of fresh leaf samples was taken from the youngest leaf samples that had completed their development, ground in liquid nitrogen, and then homogenized in 100 mM KH_2_PO_4_/0.5 mM ethylenediaminetetraacetic acid pH (7.7) buffer containing 5 mL of 1% (*w*/*v*) polyvinylpyrrolidone. Afterwards, the supernatant was shaken in a refrigerated centrifuge at 15,000× *g* for 20 min at 4 °C to separate it from the precipitate. The resulting supernatants were stored at −20 °C until use [109]. Using the Bradford technique, the total soluble proteins in leaf tissues were calculated [110]. Specific enzyme activities were calculated by normalizing the enzyme activity (expressed in enzyme units per minute) to the total soluble protein content and expressed as EU mg^−1^ protein and were calculated according to the formulas below.

Enzyme unit (EU) = (Absorbance × Volume of Assay × Dilution factor)/(Extinction coefficient × Volume of enzyme × time)

Specific activity (EU mg^−1^ protein) = EU/mg protein

CAT activity was assessed for 3 min based on the reduction in the absorbance value at 240 nm (=0.0436 mM^−1^ cm^−1^) as a result of the interaction of hydrogen peroxide with the enzyme [111].

The rate of ascorbate oxidation at 290 nm was measured to assess APX activity. This was done by monitoring the decrease in absorbance of ascorbate (ε: 2.8 mM^−1^ cm^−1^) at 290 nm over a period of 3 min [112].

Total SOD activity was assayed using the photochemical nitroblue tetrazolium (NBT) method, which measures the suppression of superoxide-mediated NBT reduction by enzymes. The Giannopolitis and Ries (1977) [113]. The technique was used to evaluate the SOD enzyme’s suppression of the photochemical reduction reaction of NBT with superoxide radicals to the blue-colored formazone at 560 nm.

GR activity was observed by monitoring the decline in absorbance at 340 nm caused by Nicotinamide adenine dinucleotide phosphate (NADPH) oxidation. During 3 min, spectrophotometric data were collected at 340 nm at the kinetic rate. The molar absorption coefficient (ε) of NADPH at 340 nm was utilized as 6.22 mM^−1^ cm^−1^ in the enzyme unit calculation [114].

The conjugation of an aromatic electrophile with a glutathione molecule is catalyzed by the GST activity. 1-chloro-2,4-dinitrobenzene (CDNB), an aromatic electrophile, is the most often utilized. Spectrophotometric measurements were performed at Kinetics rate at 340 nm for 3 min. The enzyme unit was determined using the molar absorption coefficient (ε) of CDNB at 340 nm, which was 9.6 mM^−1^ cm^−1^ [115].

The GPX activity assay relies on guaiacol oxidation by H_2_O_2_ and was quantified by monitoring the increase in absorbance at 470 nm for 3 min due to tetraguaiacol formation. The extinction coefficient was used to determine the peroxidase activity (ε: 26.6 mM^−1^ cm^−1^ at 470 nm) [116].

### 4.9. MDA and Proline Contents Analysis

To determine the level of MDA formed as a result of lipid peroxidation, an extinction coefficient of 155 mM^−1^ cm^−1^ was applied, and this value was expressed as µmol g^−1^ FW [97,117]. The proline content was measured according to the procedure of Bates et al. (1973) [118]. The proline concentration was determined using a standard curve that had previously been constructed and was given in µg g^−1^ FW.

### 4.10. Statistical Analysis

Two-way analysis of variance (ANOVA) was performed on the collected data to evaluate the individual and interactive effects of the two factors on the measured parameters using JMP Pro 17 statistical software, where significant differences were identified. The means were separated using Fisher’s least significant difference (LSD) test to enable precise comparisons between treatments. Due to low within-treatment variability and high experimental precision, Fisher’s LSD test was considered appropriate for detecting treatment differences following a significant ANOVA. In all figures and tables, error bars indicate the standard error of the mean. Means followed by different letters are significantly different based on Fisher’s LSD multiple range test.

Pearson’s correlation analysis, principal component analysis (PCA), and graphs were obtained using OriginPro, 2026. Pearson’s correlation analysis was conducted to explore the relationships among physiological, biochemical, and yield-related traits, providing insight into the degree and direction of association between variables. Additionally, PCA was performed to identify patterns and groupings among the treatments and visualize the multivariate response of the studied parameters to foliar treatments and salinity stress levels.

## 5. Conclusions

This study demonstrated that water salinity severely restricted spinach growth, yield, leaf area, and stomatal conductance at 15 dS m^−1^. While CAT, GPX, GR, GST, and SOD activities increased, APX activity decreased, indicating an enzymatic imbalance under high salt exposure. Also, it was observed that ascorbic acid, proline, and salicylic acid played complementary roles in elevating spinach tolerance to salinity by strengthening key physiological and biochemical mechanisms rather than only improving yield-related features. The application of these biostimulants most effectively recovered stomatal conductance, photosynthetic pigment performance, and antioxidant capacity, indicating interaction in regulating stress-responsive pathways. It can be concluded that proline, particularly, might alleviate these harmful effects, mainly by enhancing antioxidant defenses, preserving membrane integrity, and contributing to ion homeostasis. Salicylic acid primarily improved the K^+^/Na^+^ balance and stress signaling, whereas ascorbic acid reinforced redox buffering capacity and Ca^2+^-mediated membrane stability. In this sense, future studies could be directed to evaluate the association of biostimulants in attenuating saline stress in spinach, since they act in different but complementary tolerance processes that can maximize the plant’s response.

## Figures and Tables

**Table 1:** Effect of the application of biostimulants via foliar spray (ascorbic acid, proline, and salicylic acid) and irrigation water salinity on yield, dry weight, and leaf area in spinach.

EA	Yield (g pot^−1^)	Dry Weight (g pot^−1^)	Leaf Area (cm^2^)
C	143.4 ± 4.90 ^d^	18.5 ± 0.37 ^d^	579.3 ± 48.07 ^c^
AsA	149.1 ± 3.70 ^c^	19.4 ± 0.61 ^c^	639.1 ± 51.80 ^b^
Pro	156.3 ± 4.90 ^a^	20.2 ± 0.61 ^a^	666.7 ± 9.36 ^a^
SA	152.9 ± 5.44 ^b^	20.0 ± 0.52 ^b^	639.7 ± 37.34 ^b^
IWS			
S_0_	158.3 ± 4.78 ^b^	20.3 ± 0.59 ^c^	799.7 ± 12.24 ^b^
S_1_	165.7 ± 3.03 ^a^	21.1 ± 0.42 ^b^	937.7 ± 26.55 ^a^
S_2_	167.0 ± 6.06 ^a^	21.7 ± 0.82 ^a^	704.7 ± 52.96 ^c^
S_3_	157.8 ± 1.33 ^b^	20.5 ± 0.24 ^c^	523.8 ± 3.63 ^d^
S_4_	138.7 ± 2.21 ^c^	17.7 ± 0.32 ^d^	447.3 ± 13.35 ^e^
S_5_	115.2 ± 1.88 ^d^	16.1 ± 0.10 ^e^	374.0 ± 13.98 ^f^
EA Treatment	**	**	**
IWS Treatment	**	**	**
EA × IWS interaction	**	**	**

**: Indicates significance at the 1% level. EA: exogenous application, C: control, AsA: ascorbic acid, Pro: proline, SA: salicylic acid, IWS: irrigation water salinity, S_0_: (0.38 dS/m), S_1_: (2.0 dS/m), S_2_: (4.0 dS/m), S_3_: (7.0 dS/m), S_4_: (10.0 dS/m), S_5_: (15.0 dS/m). Values represent mean ± standard error (n:3). Different lowercase letters indicate significant differences among treatments based on the LSD test.

**Table 2:** Effect of the application of biostimulants via foliar spray (ascorbic acid, proline, and salicylic acid) and irrigation water salinity on stomatal conductance, total chlorophyll, and carotenoid in spinach.

EA	Stomatal Conductance (mmol m^−2^ s^−1^)	Total Chlorophyll (mg g^−1^ FW)	Carotenoid (mg g^−1^ FW)
C	162.50 ± 19.65 ^d^	3.55 ± 0.10 ^d^	0.60 ± 0.01 ^d^
AsA	204.72 ± 31.88 ^c^	3.67 ± 0.11 ^b^	0.63 ± 0.01 ^b^
Pro	250.22 ± 36.94 ^a^	3.85 ± 0.12 ^a^	0.65 ± 0.01 ^a^
SA	210.61 ± 28.52 ^b^	3.65 ± 0.11 ^c^	0.62 ± 0.01 ^c^
IWS			
S_0_	426.08 ± 26.90 ^a^	4.40 ± 0.05 ^a^	0.74 ± 0.00 ^a^
S_1_	279.58 ± 14.66 ^b^	4.00 ± 0.14 ^b^	0.68 ± 0.00 ^b^
S_2_	205.66 ± 24.27 ^c^	3.81 ± 0.11 ^c^	0.65 ± 0.02 ^c^
S_3_	158.83 ± 5.89 ^d^	3.59 ± 0.02 ^d^	0.61 ± 0.00 ^d^
S_4_	99.75 ± 5.31 ^e^	3.26 ± 0.03 ^e^	0.57 ± 0.00 ^e^
S_5_	72.16 ± 3.84 ^f^	3.01 ± 0.02 ^f^	0.50 ± 0.00 ^f^
EA Treatment	**	**	**
IWS Treatment	**	**	**
EA × IWS interaction	**	**	**

**: Indicates significance at the 1% level. EA: exogenous application, C: control, AsA: ascorbic acid, Pro: proline, SA: salicylic acid, IWS: irrigation water salinity, S_0_: (0.38 dS/m), S_1_: (2.0 dS/m), S_2_: (4.0 dS/m), S_3_: (7.0 dS/m), S_4_: (10.0 dS/m), S_5_: (15.0 dS/m). Values represent mean ± standard error (n:3). Different lowercase letters indicate significant differences among treatments based on the LSD test.

**Table 3:** Effect of the application of biostimulants via foliar spray (ascorbic acid, proline, and salicylic acid) and irrigation water salinity on catalase (CAT), ascorbate peroxidase (APX), superoxide dismutase (SOD), guaiacol peroxidase (GPX), glutathione reductase (GR), and glutathione s-transferase (GST) in spinach.

EA	CAT (EU mg ^−1^ Protein)	APX	SOD	GPX	GR	GST
C	400.75 ± 19.11 ^d^	1.69 ± 0.14 ^d^	45.95 ± 1.51 ^d^	0.50 ± 0.03 ^c^	0.33 ± 0.02 ^c^	0.55 ± 0.02 ^d^
AsA	535.53 ± 20.21 ^b^	2.10 ± 0.08 ^c^	49.05 ± 2.96 ^c^	0.62 ± 0.03 ^b^	0.34 ± 0.03 ^c^	0.62 ± 0.05 ^c^
Pro	628.73 ± 22.09 ^a^	2.56 ± 0.22 ^a^	57.14 ± 2.89 ^a^	0.66 ± 0.02 ^a^	0.55 ± 0.03 ^a^	0.70 ± 0.05 ^a^
SA	487.01 ± 19.25 ^c^	2.32 ± 0.16 ^b^	52.88 ± 2.58 ^b^	0.50 ± 0.01 ^c^	0.48 ± 0.03 ^b^	0.64 ± 0.02 ^b^
IWS						
S_0_	387.20 ± 21.47 ^f^	3.25 ± 0.22 ^a^	51.07 ± 4.39 ^c^	0.36 ± 0.02 ^f^	0.25 ± 0.02 ^f^	0.41 ± 0.02 ^f^
S_1_	446.11 ± 24.61^e^	2.54 ± 0.09 ^b^	54.26 ± 1.68 ^b^	0.49 ± 0.01 ^e^	0.34 ± 0.03 ^e^	0.53 ± 0.02 ^e^
S_2_	486.49 ± 33.40 ^d^	2.25 ± 0.15 ^c^	57.94 ± 3.19 ^a^	0.56 ± 0.04 ^d^	0.39 ± 0.02 ^d^	0.56 ± 0.05 ^d^
S_3_	550.90 ± 26.62 ^c^	2.01 ± 0.13 ^d^	54.69 ± 3.80 ^b^	0.61 ± 0.02 ^c^	0.44 ± 0.02 ^c^	0.63 ± 0.00 ^c^
S_4_	585.55 ± 24.31 ^b^	1.64 ± 0.08 ^e^	44.92 ± 2.76 ^d^	0.66 ± 0.03 ^b^	0.49 ± 0.02 ^b^	0.76 ± 0.03 ^b^
S_5_	621.77 ± 27.87 ^a^	1.31 ± 0.06 ^f^	44.65 ± 2.85 ^d^	0.74 ± 0.03 ^a^	0.63 ± 0.03 ^a^	0.89 ± 0.04 ^a^
EA Treatment	**	**	**	**	**	**
IWS Treatment	**	**	**	**	**	**
EA × IWS interaction	**	**	**	**	**	**

**: Indicates significance at the 1% level. EA: exogenous application, C: control, AsA: ascorbic acid, Pro: proline, SA: salicylic acid, IWS: irrigation water salinity, S_0_: (0.38 dS/m), S_1_: (2.0 dS/m), S_2_: (4.0 dS/m), S_3_: (7.0 dS/m), S_4_: (10.0 dS/m), S_5_: (15.0 dS/m). Values represent mean ± standard error (n:3). Different lowercase letters indicate significant differences among treatments based on the LSD test.

**Table 4:** Effect of the application of biostimulants via foliar spray (ascorbic acid, proline, and salicylic acid) and irrigation water salinity on malondialdehyde (MDA) and proline in spinach.

EA	MDA (µmol g^−1^ FW)	Proline (µg g^−1^ FW)
C	0.74 ± 0.02 ^a^	6.66 ± 1.29 ^d^
AsA	0.65 ± 0.02 ^c^	8.82 ± 1.51 ^b^
Pro	0.60 ± 0.00 ^d^	9.94 ± 1.74 ^a^
SA	0.68 ± 0.02 ^b^	7.55 ± 1.13 ^c^
IWS		
S_0_	0.57 ± 0.01 ^f^	3.33 ± 0.15 ^f^
S_1_	0.60 ± 0.01 ^e^	4.03 ± 0.17 ^e^
S_2_	0.64 ± 0.02 ^d^	4.79 ± 2.17 ^d^
S_3_	0.66 ± 0.01 ^c^	6.48 ± 0.45 ^c^
S_4_	0.72 ± 0.01 ^b^	10.73 ± 0.69 ^b^
S_5_	0.80 ± 0.02 ^a^	20.05 ± 0.87 ^a^
EA Treatment	**	**
IWS Treatment	**	**
EA × IWS interaction	**	**

**: Indicates significance at the 1% level. EA: exogenous application, C: control, AsA: ascorbic acid, Pro: proline, SA: salicylic acid, IWS: irrigation water salinity, S_0_: (0.38 dS/m), S_1_: (2.0 dS/m), S_2_: (4.0 dS/m), S_3_: (7.0 dS/m), S_4_: (10.0 dS/m), S_5_: (15.0 dS/m). Values represent mean ± standard error (n:3). Different lowercase letters indicate significant differences among treatments based on the LSD test.

**Table 5:** Effect of the application of biostimulants via foliar spray (ascorbic acid, proline, and salicylic acid) and irrigation water salinity on malondialdehyde in spinach ionic homeostasis.

EA	Na^+^ (%)	K^+^ (%)	Ca^2+^ (%)	K/Na (%)	Ca/Na (%)
C	1.28 ± 0.12 ^a^	4.32 ± 0.13 ^d^	0.93 ± 0.05 ^d^	4.48 ± 0.73 ^d^	1.01 ± 0.18 ^d^
AsA	1.13 ± 0.11 ^c^	5.01 ± 0.09 ^b^	1.22 ± 0.07 ^a^	5.37 ± 0.60 ^b^	1.38 ± 0.19 ^a^
Pro	1.18 ± 0.09 ^b^	4.88 ± 0.12 ^c^	1.00 ± 0.08 ^c^	5.17 ± 0.75 ^c^	1.16 ± 0.23 ^c^
SA	1.09 ± 0.09 ^d^	5.09 ± 0.12 ^a^	1.04 ± 0.11 ^b^	5.67 ± 0.67 ^a^	1.30 ± 0.25 ^b^
IWS					
S_0_	0.53 ± 0.15 ^f^	5.57 ± 0.08 ^a^	1.57 ± 0.07 ^a^	10.53 ± 0.24 ^a^	2.95 ± 0.09 ^a^
S_1_	0.80 ± 0.02 ^e^	5.30 ± 0.07 ^b^	1.32 ± 0.04 ^b^	6.75 ± 0.24 ^b^	1.68 ± 0.09 ^b^
S_2_	0.99 ± 0.14 ^d^	4.90 ± 0.14 ^c^	1.02 ± 0.07 ^c^	5.05 ± 0.67 ^c^	1.07 ± 0.18 ^c^
S_3_	1.35 ± 0.04 ^c^	4.62 ± 0.08 ^d^	0.95 ± 0.06 ^d^	3.47 ± 0.15 ^d^	0.72 ± 0.06 ^d^
S_4_	1.54 ± 0.03 ^b^	4.42 ± 0.09 ^e^	0.75 ± 0.04 ^e^	2.90 ± 0.12 ^e^	0.49 ± 0.03 ^e^
S_5_	1.80 ± 0.03 ^a^	4.16 ± 0.12 ^f^	0.67 ± 0.01 ^f^	2.33 ± 0.09 ^f^	0.37 ± 0.01 ^f^
EA Treatment	**	**	**	**	**
IWS Treatment	**	**	**	**	**
EA × IWS interaction	**	**	**	**	**

**: Indicates significance at the 1% level. EA: Exogenous application, C: Control, AsA: Ascorbic acid, Pro: Proline, SA: Salicylic acid, IWS: Irrigation water salinity, S_0_: (0.38 dS/m), S_1_: (2.0 dS/m), S_2_: (4.0 dS/m), S_3_: (7.0 dS/m), S_4_: (10.0 dS/m), S_5_: (15.0 dS/m). Values represent mean ± standard error (n:3). Different lowercase letters indicate significant differences among treatments based on the LSD test.

## Data Availability

All original findings generated in this study are presented within the article. Additional information or clarification may be obtained by contacting the corresponding author.
